# A Replisome’s journey through the bacterial chromosome

**DOI:** 10.3389/fmicb.2015.00562

**Published:** 2015-06-05

**Authors:** Thomas R. Beattie, Rodrigo Reyes-Lamothe

**Affiliations:** Department of Biology, McGill University, Montreal, QC, Canada

**Keywords:** DNA replication, replisome, bacteria, chromosome, evolution, DNA polymerase, *Escherichia coli*, *Bacillus subtilis*

## Abstract

Genome duplication requires the coordinated activity of a multi-component machine, the replisome. In contrast to the background of metabolic diversity across the bacterial domain, the composition and architecture of the bacterial replisome seem to have suffered few changes during evolution. This immutability underlines the replisome’s efficiency in copying the genome. It also highlights the success of various strategies inherent to the replisome for responding to stress and avoiding problems during critical stages of DNA synthesis. Here we summarize current understanding of bacterial replisome architecture and highlight the known variations in different bacterial taxa. We then look at the mechanisms in place to ensure that the bacterial replisome is assembled appropriately on DNA, kept together during elongation, and disassembled upon termination. We put forward the idea that the architecture of the replisome may be more flexible that previously thought and speculate on elements of the replisome that maintain its stability to ensure a safe journey from origin to terminus.

## The Architecture of the Bacterial Replisome

Replication of chromosomal DNA is fundamental to the propagation of all bacterial species. This essential process faces many complex mechanistic challenges. Parental DNA must be unwound, and the resulting template strands replicated simultaneously with great efficiency and accuracy. Furthermore, because these two template strands have opposing polarity, the two new DNA strands are synthesized differently, with one strand—the leading strand—synthesized continuously, and the other—the lagging strand—synthesized as a series of short Okazaki fragments. A multi-protein complex known as the replisome has evolved to coordinate all the core enzymatic activities required for these coupled processes into a single molecular machine.

### Replisome Structure in *E. coli*

**FIGURE 1:**
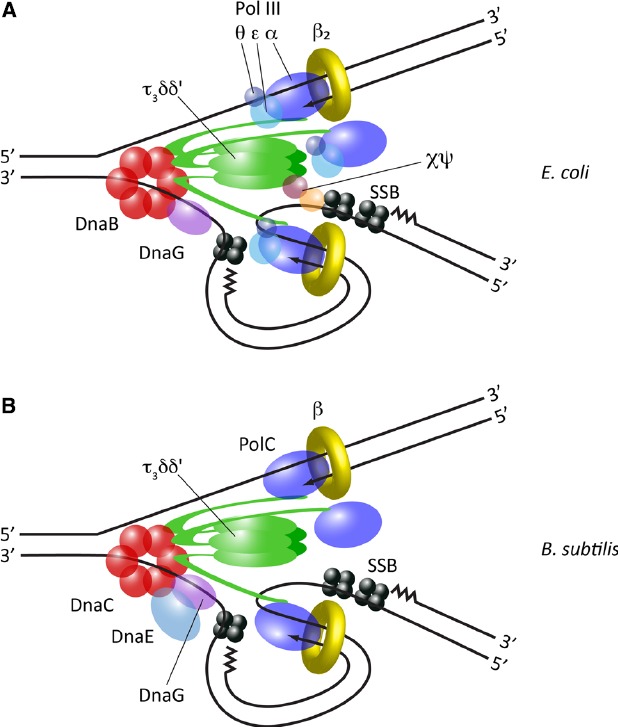
**Replisome architecture in bacteria. (A)** Architecture of the *E. coli* replisome, derived from *in vitro* studies and direct observation *in vivo*. **(B)** Architecture of the *B. subtilis* replisome, predominantly derived from *in vitro* reconstitution studies.

Replisome structure is currently best understood in the Gram-negative bacterium *Escherichia coli*. Decades of genetic and biochemical research have enabled all the essential components of the replisome in this organism to be identified, and a series of protein-protein interactions which link them into a single entity to be mapped. Indeed, it is possible to reconstitute a fully functional replisome *in vitro* from purified *E. coli* proteins (Wu et al., 1992). At the core of the replisome is the DnaB homohexameric helicase, which encircles single-stranded DNA (ssDNA) on the lagging strand and unwinds the parental DNA duplex. Copying of the resulting template strands is performed by DNA polymerase III (Pol III), which consists of a catalytic subunit α, a proofreading exonuclease subunit ε, and a poorly conserved non-essential subunit θ. The exact function of θ is still unclear, but it may moderately stimulate the exonuclease activity of ε (Slater et al., 1994). To achieve the processivity needed to synthesize the entire chromosome, Pol III associates with the dimeric β sliding clamp, which is assembled around DNA by the pentameric τ_3_δδ′ clamp loader complex. Crucially, both DnaB and Pol III α directly interact with the C-terminal domain of τ, and thus the clamp loader additionally provides an architectural function, physically coupling template unwinding with DNA synthesis (Figure 1A).

It was previously assumed that only two Pol III complexes were present within the replisome, divided between the leading and lagging strands. However, elegant *in vitro* reconstitution experiments surprisingly demonstrated that the *E. coli* replisome can incorporate three Pol III complexes, multimerised by the trimeric τ within the clamp loader (McInerney et al., 2007). More recently, an unprecedented level of detail has been obtained by directly visualizing individual components of the replisome in living *E. coli* cells by fluorescence microscopy (Reyes-Lamothe et al., 2010). This work has confirmed the presence of three active polymerases within a single replisome. Further *in vitro* studies have suggested the third replicative polymerase may serve as a backup to support efficient synthesis of the lagging strand (Georgescu et al., 2011).

A trimeric polymerase architecture was previously unanticipated because the clamp loader was thought to consist of τ_2_γδδ′, γ being an alternative C-terminally truncated form of τ which is unable bind to Pol III and derives from a frameshift during translation of the dnaX gene. The role of γ is now unclear; like *E. coli*, clamp loader complexes from a number of other bacterial species appear to possess a τ-only structure (Bruck and O’Donnell, 2000; Bruck et al., 2002, 2005; Bullard et al., 2002; Jarvis et al., 2005a; Sanders et al., 2010). However, although the generation of γ by a frameshift mechanism appears to be limited to enterobacteria (Blinkova et al., 1997), γ-like subunits have been detected in *Thermus thermophilus* and *Caulobacter crescentus*, and furthermore these arise from distinct mechanisms for truncating the dnaX gene product—transcriptional slippage and controlled proteolysis, respectively (Yurieva et al., 1997; Vass and Chien, 2013). This hints at convergent evolution for generating γ, suggesting that in at least some bacterial species a clamp loader containing this truncated product may play a specialized role. It has been suggested for example that γ may be important in dealing with DNA damage (Reyes-Lamothe et al., 2010; Vass and Chien, 2013), but its precise role remains to be elucidated.

Multimerisation of Pol III within the *E. coli* replisome presents a potential topological problem as polymerases must track in opposite directions on the leading and lagging strands, but yet are arranged symmetrically through their interaction with the clamp loader. The replisome has evolved to ensure that DNA unwinding and synthesis proceed uninterrupted by allowing the transient accumulation of ssDNA loops between Pol III and the helicase on the lagging strand. These loops are rapidly bound by the tetrameric ssDNA binding protein, SSB, which protects them from damage. The cyclical assembly, growth and disassembly of these loops, which accompanies the synthesis of each Okazaki fragment, has been compared to the slide of a trombone (trombone model; Sinha et al., 1980). The presence of such trombone loops has been directly observed in simpler bacteriophage replisomes by electron microscopy and single-molecule biophysical studies (Park et al., 1998; Hamdan et al., 2009).

Initiation of each Okazaki fragment requires the synthesis of an RNA primer, which is subsequently extended by Pol III following loading of the β clamp onto the primer-template junction. In *E. coli* this is performed by the DnaG primase, which interacts directly with DnaB, thus facilitating primer deposition on the emerging lagging strand template. However, biochemical studies suggest that DnaG is not a constitutive component of the replisome, but rather associates with DnaB transiently; indeed it has been suggested that each primer is synthesized by a new DnaG molecule recruited from solution (Wu et al., 1992). In contrast, biophysical measurements on bacteriophage T4 replisomes have shown that primase behavior is stochastic, sometimes dissociating from the helicase following primer synthesis, but sometimes remaining tightly bound (Manosas et al., 2009). Unlike other replisome components, it has so far not been possible to observe DnaG *in vivo* by microscopy. It will be interesting in the future to directly determine the nature of the DnaG-DnaB interaction in its native environment of the living cell.

Since the predicted number of Okazaki fragments generated during chromosomal replication in *E. coli* exceeds the number of β clamps available in the cell, these clamps must be reused throughout replication (Leu et al., 2000). While some *in vitro* data suggests that clamps may be immediately recycled with Pol III between successive Okazaki fragments (Tanner et al., 2011), live cell microscopy data from both *E. coli* and *Bacillus subtilis* has demonstrated that clamps accumulate on DNA behind the replisome (Su’etsugu and Errington, 2011; Moolman et al., 2014). This therefore supports an alternative model whereby clamps are reloaded from a pool left on the lagging strand. Such an accumulation of β clamps may serve as an important landing platform on DNA for the recruitment of DNA modifying enzymes following the passage of the replisome. Indeed, it has even been proposed that it is for this function, rather than their role as processivity factors, that clamp molecules originally evolved (Georgescu et al., 2015).

### Replisome Structure in *B. subtilis*

To date, the replisome has only been characterized in detail in one organism other than *E. coli*: the low-GC Gram-positive bacterium *Bacillus subtilis* (Figure 1B and Table 1). A fully functional replisome with physiological rates of coupled leading and lagging strand synthesis has been reconstituted *in vitro* from purified *B. subtilis* proteins, defining its minimal protein requirements (Sanders et al., 2010). This study demonstrated a conserved core replisome structure with *E. coli*, consisting of a homohexameric helicase (DnaC) which interacts with both a distributive primase (DnaG) and a pentameric τ_3_δδ′ clamp loader complex. Replication is also similarly dependent on a dimeric β clamp and SSB.

**TABLE 1:** **A comparison of replication protein nomenclature in*E. coli* and *B. subtilis***.

**Protein function**	***E. coli***	***B. subtilis***
Origin activator	DnaA	DnaA
Helicase loader	DnaC	DnaI
Helicase	DnaB	DnaC
Primase	DnaG	DnaG
Clamp loader	τ_3_δδ′χψ	τ_3_δδ′
Primary replicative polymerase	Pol III (αεθ)	PolC
Accessory replicative polymerase	–	DnaE
ssDNA binding protein	SSB	SSB
Sliding clamp	β_2_	β_2_

However, the *B. subtilis* replisome also exhibits some notable differences compared to *E. coli*. Two distinct C-type DNA polymerases—DnaE and PolC—are required for chromosomal replication in this organism (Dervyn et al., 2001), a feature common to many species of Gram-positive bacteria (Timinskas et al., 2014). DnaE is related to *E. coli* Pol III α, while PolC is significantly different in domain organization and additionally possesses an intrinsic exonuclease activity. Initial genetic analysis suggested that the activities of PolC and DnaE may be divided between the leading and lagging strands respectively (Dervyn et al., 2001), but more recent biochemical analysis suggests that DnaE may in fact purely be used to extend RNA primers a short distance on the lagging strand, before PolC rapidly displaces it to synthesize the majority of DNA on both lagging and leading strands (Sanders et al., 2010). This division of function may be enforced by differences in the mode of interaction of the two polymerases with other components of the replisome; while PolC is linked to the helicase via τ, as in *E. coli* (Bruck and O’Donnell, 2000), it has been reported that DnaE interacts directly with the helicase and DnaG, forming a “primosome” complex (Rannou et al., 2013).

A mechanism of primer extension through the sequential action of two distinct DNA polymerases is also used in eukaryotic organisms. However, it has been noted that this mechanism is inherently wasteful, as the short stretch of error-prone DNA synthesized by the first DNA polymerase is entirely removed later in replication anyway (Forterre, 2013). This is also likely to be the case in *B. subtilis* since DnaE lacks a proofreading activity, although it is unclear which polymerase replaces the DNA synthesized by DnaE; it is possible that DNA Pol I fulfils this function as it removes RNA primers prior to Okazaki fragment ligation. Since the respective proteins involved in lagging strand synthesis in eukaryotes and bacteria are non-orthologous this dual polymerase mechanism likely arose through convergent evolution. At present however, it is still unclear what selective advantage, if any, DnaE-PolC coordination on the lagging strand provides.

While the core replisome components have been identified in *B. subtilis*, their relative stoichiometry within the complex has yet to be fully determined. In particular it is unknown how many polymerases are typically present within the *B. subtilis* replisome. The role of PolC on both leading and lagging strands suggests that at least two molecules of this polymerase must be simultaneously present within the replisome. Furthermore, given the interaction between PolC and the trimeric τ of the clamp loader it is possible that three molecules of PolC may be present, similar to *E. coli* Pol III. Rapid displacement of non-proofreading DnaE by PolC on the lagging strand is presumably important for genome integrity, and therefore it may be speculated that maintaining a high relative local concentration of PolC within the replisome would be a mechanism for achieving this. It should be noted that the interaction between τ and PolC is significantly weaker than the equivalent interaction in *E. coli* (Bruck and O’Donnell, 2000). It is currently unclear whether this represents a physiological property of the *B. subtilis* replisome with implications for its architecture or is due to the absence of other stabilizing components that have yet to be identified. Further characterisation of the *B. subtilis* replisome both *in vitro* and *in vivo* will be important for determining the precise structure of the replisome in this organism and how its multiple polymerases are efficiently coordinated.

Notably, an analysis of polymerase distribution across bacterial genomes has demonstrated that a third major combination of replicative polymerases is found in some organisms: PolC together with a DnaE distinct from that of both *E. coli* and *B. subtilis* (Timinskas et al., 2014). The DNA replication machinery has yet to be examined in any of these species, and it will be interesting in the future to determine if their replisomes employ similar or different strategies to those already described for coordinating multiple DNA polymerases.

### Replisome Structure in Other Bacterial Organisms

Despite the extreme diversity present across the bacterial kingdom in many areas of cell biology, a relatively high degree of conservation has been noted for many DNA replication proteins, highlighting the central importance of this cellular process. However, it is not clear whether the assembly of these proteins into a higher order replisome complex also follows a conserved architecture. Beyond *E. coli* and *B. subtilis* this question has not been extensively addressed.

Replisome subassemblies have been successfully reconstituted using purified proteins from *Aquifex aeolicus* (Bruck et al., 2002), *Streptococcus pyogenes* (Bruck and O’Donnell, 2000), *Staphylococcus aureus* (Bruck et al., 2005), *Thermus thermophilus* (Bullard et al., 2002), and *Pseudomonas aeruginosa* (Jarvis et al., 2005a). These studies demonstrate that the core replisome structure of a τ_3_δδ′ clamp loader linked to the catalytic subunit of the replicative polymerase is likely to be a conserved feature among bacteria. In the case of *T. thermophilus* the clamp loader can also incorporate γ, although the same caveats should apply as in *E. coli*. However, the organisms examined to date still constitute a tiny sample of total bacterial diversity. Furthermore, it is still unclear if the mode of interaction between this replisome core and other components such as the helicase, primase, and additional polymerases is similarly conserved.

For example, two additional clamp loader subunits, χ and ψ, are present in *E. coli*. While not essential, χ and ψ are required for normal growth. These two components form a tight dimer and interact with both τ and SSB, resulting in both the stabilization of the clamp loader complex and the replisome overall (Olson et al., 1995; Marceau et al., 2011). The χψ subassembly has also been proposed to play a role in promoting the access of Pol III to newly synthesized primers (Yuzhakov et al., 1999). However, sequence analysis suggests these two subunits are only present in proteobacteria, and it is unclear what, if anything, fulfils their replisome function in other bacterial lineages. It should be noted that in *P. aeruginosa* a ψ subunit was identified through its co-purification with χ despite it being unidentifiable on the basis of sequence homology alone (Jarvis et al., 2005b). This raises the possibility that highly diverged functional homologs of these subunits may indeed exist in other organisms where they have not yet been detected, and highlights the importance of empirical research to describing replisome composition across the bacterial kingdom.

*In vivo* characterisation of replisome architecture is still lacking outside of *E. coli*. Tools are gradually being developed to study the DNA replication machinery in living cells of other species, such as *Helicobacter pylori* (Sharma et al., 2014), *Mycobacterium smegmatis* (Santi and McKinney, 2015; Trojanowski et al., 2015), and *B. subtilis* (Lemon and Grossman, 1998), and it is hoped that in the future determining replisome stoichiometries *in vivo* will enable a better assessment of replisome structure conservation.

## Mechanisms of Replisome Assembly

Assembly of the bacterial replisome is a tightly regulated process. DNA replication is normally initiated at a specific origin locus by the assembly of just two replisomes, each of which synthesizes half of the circular chromosome. It is important to restrict replisome assembly beyond this specific initiation event, to prevent over-replication of DNA and chromosome instability. Different mechanisms exist to ensure specificity in replisome assembly, but they appear to converge at the level of controlling the loading of the DnaB helicase onto ssDNA (Fang et al., 1999). Once this loading step has been overcome, DnaB can serve as the platform upon which the rest of the replisome is assembled, through its direct interactions with the primase and clamp loader. Interestingly, a similar strategy is observed in eukaryotic organisms, despite the use of completely non-orthologous proteins, whereby loading and activation of the replicative helicase Mcm2-7 is also the limiting step in replisome assembly (Yeeles et al., 2015).

### Replisome Assembly During Initiation

**FIGURE 2:**
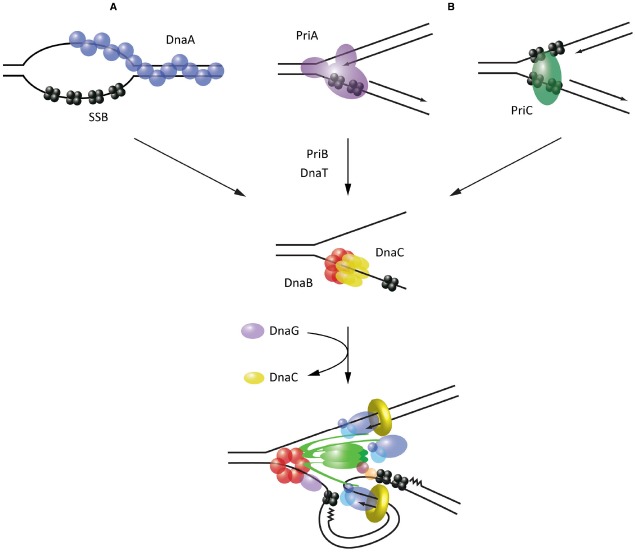
**Mechanisms of helicase loading leading to replisome assembly in *E. coli*. (A)** Recognition and melting of the *oriC* locus during initiation by DnaA. **(B)** Recognition of abandoned fork structures during replisome reloading by PriA and PriC. All pathways converge on the loading of the replicative helicase DnaB, which acts as an assembly platform for the remaining replisome components.

Initiation of replication is best understood in *E. coli*, where replication originates from the *oriC* locus. *oriC* is recognized and melted through sequence-specific binding of the AAA+ protein, DnaA (Leonard and Grimwade, 2011). The prevailing current model proposes that DnaA oligomerizes into a helical filament, around which bound *oriC* DNA is wrapped (Erzberger et al., 2006). This destabilizes the neighboring duplex unwinding element (DUE), leading to DNA melting as the DnaA filament extends onto transiently exposed ssDNA (Duderstadt et al., 2011). The resulting bubble of SSB-coated ssDNA is a substrate for the assembly of two DnaB helicases (Fang et al., 1999). Crucially, however, DnaB loading onto such structures is not spontaneous; it is restricted to the *oriC* locus through a dependence on the helicase loader protein, DnaC (Figure 2A).

DnaC has been shown to interact with both DnaA and DnaB, and thus serves as link to recruit DnaB to melted origins (Mott et al., 2008). Furthermore, it appears to play an active role in loading the helicase. Structural analysis of DnaC has shown that this protein resembles DnaA and similarly adopts a helical filament when bound to ssDNA (Mott et al., 2008). More recently, it has been demonstrated that this spiral configuration enables DnaC to break open the DnaB hexameric ring as the two proteins interact, allowing ssDNA to enter the central chamber of the helicase (Arias-Palomo et al., 2013). Association of DnaG primase with DnaB triggers the release of DnaC and thus activates the helicase, beginning the process of replisome assembly (Davey et al., 2002; Makowska-Grzyska and Kaguni, 2010). Interestingly, it has been shown that while two DnaB helicases must be loaded at *oriC* (Fang et al., 1999), the first of these is always deposited on the lower strand (Weigel and Seitz, 2002). A model for asymmetric loading of DnaB has thus been proposed, whereby DnaC-DnaB on one strand is loaded via the interaction of DnaC with DnaA, while on the other strand DnaC-DnaB is loaded via a direct interaction observed between DnaB and DnaA (Mott et al., 2008). Such a loading mechanism is attractive because it accounts for the need for the two helicases—and their associated replisomes—to proceed bidirectionally from *oriC*.

This helicase loading pathway may not be well conserved across bacterial organisms. *B. subtilis* for example possesses orthologs of DnaA, DnaC (called DnaI) and DnaB (called DnaC) but also requires two additional proteins for helicase loading that have no orthologs in *E. coli*: DnaB, and DnaD (Smits et al., 2010). The precise role of these additional proteins is still unclear. Furthermore, despite being related to *E. coli* DnaC, the *B. subtilis* helicase loader DnaI may employ a different mechanism to load the helicase on DNA; structural and biochemical data suggest that rather than acting as a ring-opener, DnaI may act as a template for the assembly of the hexameric helicase around ssDNA from individual monomers (Velten et al., 2003; Liu et al., 2013). It has been proposed from sequence analysis that some bacterial species including *A. aeolicus* posses a helicase loader distinct from that present in either *E. coli* or *B. subtilis* (Robinson et al., 2012). However, a low-resolution structural comparison suggests that *E. coli* and *A. aeolicus* DnaC form similar spiral oligomers and therefore may employ the same mechanism for opening the DnaB hexameric ring (Arias-Palomo et al., 2013).

Helicase loading may differ even further in other bacterial organisms, since many species lack an identifiable DnaC homolog. For example, helicases from *Pseudomonas* species can be loaded *in vitro* in the absence of a loader protein, in contrast to *E. coli* (Caspi et al., 2001). Similarly, the helicase from *H. pylori* is sufficient to complement *E. coli* lacking DnaC, suggesting it is functionally loaded onto DNA without a loader (Soni et al., 2005). Interestingly, this correlates with the ability of this helicase to uniquely form a double hexamer (Stelter et al., 2012). Thus it may be speculated that in some bacterial species at least, the replicative helicase possesses structural adaptations that confer intrinsic self-loading ability. It is unclear how site-specific loading would be maintained in such cases.

### Replisome Assembly Following Fork Collapse

It is now apparent that replication fork structures can frequently break down during chromosomal replication, leading to collapse of the replisome. This is especially the case under stress conditions, when lesions in DNA become more prevalent, but is also likely to be the case during unperturbed replication in healthy cells (Maisnier-Patin et al., 2001). Thus to ensure complete replication of the chromosome, there is a requirement for systems that can efficiently reload replisomes at sites where they have collapsed. Crucially, these mechanisms must be specific enough to ensure that replisomes aren’t simply loaded indiscriminately onto any ssDNA structure.

In *E. coli*, this reloading process is restricted to abandoned replication forks through the activity of two proteins, PriA and PriC, which recognize specific DNA structures (Figure 2B). PriA possesses a modular arrangement of DNA-binding domains which allow it to specifically bind the three arms of forked DNA structures, with a preference for those possessing a fully extended leading strand (Heller and Marians, 2005a; Bhattacharyya et al., 2014). PriC provides complementary activity by recognizing fork structures lacking a leading strand; these can arise when the leading strand polymerase stalls at a lesion and becomes uncoupled from the continuing lagging strand polymerase (Heller and Marians, 2005a). PriC probably recognizes such fork structures through an interaction with SSB (Wessel et al., 2013). In some cases the presence of SSB may rely on prior unwinding of the lagging strand by Rep or even PriA (Heller and Marians, 2005b).

Supported by genetic analysis, reconstitution of helicase reloading *in vitro* has demonstrated that these different modes of substrate recognition lead to two distinct pathways of helicase reloading. PriA substrate recognition leads to the sequential recruitment of PriB, DnaT and finally the DnaC-DnaB complex for loading (Lopper et al., 2007). Alternatively, substrate recognition by PriC leads directly to the recruitment of DnaC-DnaB (Heller and Marians, 2006). Notably, while the components of these pathways have been characterized extensively, the precise mechanisms that lead to the recruitment of DnaC and loading of the helicase specifically onto the lagging strand, are still unknown. Intriguingly, DnaT has been suggested from structural studies to form a helical filament on DNA (Liu et al., 2014), raising the possibility that it could act analogously to DnaA in recruiting DnaC. The mode of interaction between PriC and DnaC-DnaB has yet to be characterized, although high-throughput studies have identified a potential interaction between PriC and DnaB (Butland et al., 2005).

Notably, many bacteria lack an ortholog of *E. coli* PriC and thus it is unclear what, if anything, fulfils its function in these species. Furthermore, while PriA does seem to be relatively well-conserved, its mode of action may not be; reconstitution of helicase reloading in *B. subtilis* suggests that PriA acts to recruit the helicase through the DnaD, DnaB and DnaI proteins used during initiation, rather than a specialized set of proteins as in *E. coli* (Manhart and McHenry, 2013). The exact mechanism of replisome assembly both at origins of replication and following replication fork collapse may therefore differ significantly between bacterial species. Nonetheless, it appears that these different pathways converge on the limiting step of loading of the replicative helicase, which serves as the core replisome component upon which other replication proteins are assembled.

## Dynamics and Stability of the Replisome

Looking at the replisome in action must surely be like looking at a beehive. Like bees performing their collection duties, multiple replisome components are continuously arriving and leaving with every cycle of synthesis on the lagging strand. One new copy of primase and the β clamp dimer is needed to start every Okazaki fragment, and multiple copies of SSB are recruited and displaced as a result of DNA melting by helicase and synthesis by Pol III respectively (Wu et al., 1992).

A long-standing debate on the coordination of replisome activities has been over how Pol III, evolved for high processivity, frequently dissociates from the lagging strand to ensure efficient cycles of Okazaki fragment synthesis. The collision model hypothesizes that Pol III dissociates upon encountering the previous Okazaki fragment. It has been suggested that Pol III senses elimination of template ssDNA as it approaches the end of each Okazaki fragment, which weakens its affinity for the β clamp through an unknown mechanism, thus decreasing the stability of the polymerase on DNA (Georgescu et al., 2009). In contrast, the signaling model proposes that a specific signal intrinsic to the replisome triggers Pol III dissociation following synthesis of each new primer (Wu et al., 1992). This is supported by the fact that Pol III dissociation rates following collision *in vitro* are insufficient to support the rate of synthesis required during chromosomal replication—at least in the context of a dimeric Pol III replisome (Dohrmann et al., 2011). Recent single-molecule data has suggested that the source of this dissociation signal may be the accumulation of topological stress at the replication fork as the physically coupled polymerases track around helical DNA during synthesis (Kurth et al., 2013). Although their relative importance is still contested, some *in vitro* experiments have suggested that in fact both mechanisms operate redundantly to maximize the efficiency of Okazaki fragment synthesis (Li and Marians, 2000; Hamdan et al., 2009).

In contrast with this dynamic picture, the replisome also needs to be a very stable assembly to accomplish replication of the long chromosomal DNA molecules. In *E. coli*, chromosome duplication takes the replisome over forty minutes of continuous work even at synthesis rates of 1 kb per second (Kubitschek and Newman, 1978; Kornberg and Baker, 1992). The importance of keeping the replisome active and in one piece is demonstrated by the observation that the majority of double strand breaks, which are potentially lethal lesions to the cell, occur during DNA replication (Cox et al., 2000; Pennington and Rosenberg, 2007) often as a result of extended pauses in replication fork progression (Michel et al., 2004). Judging by relatively infrequent replisome collapse events—once every five generations in *E. coli* (Maisnier-Patin et al., 2001)—high stability seems to be inherent to the replisome.

**FIGURE 3:**
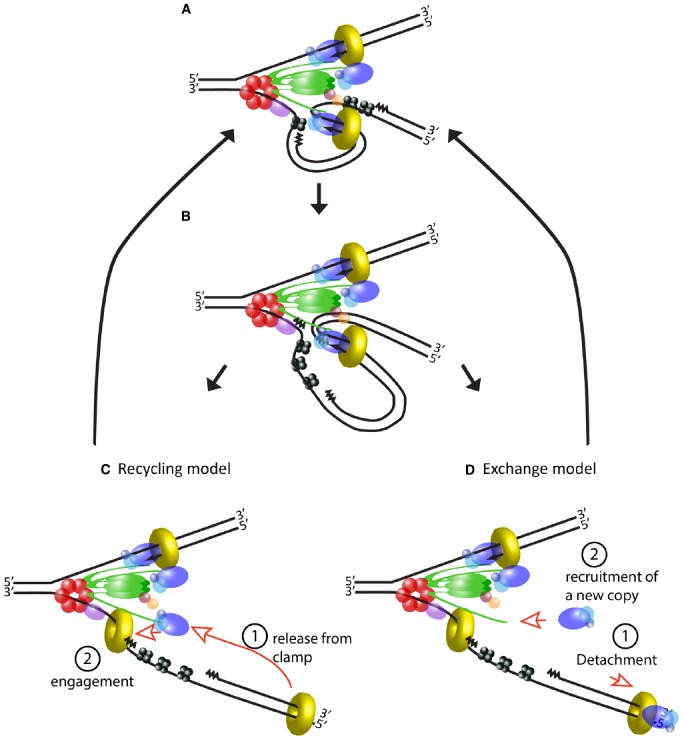
**Usage of DNA polymerase during lagging strand synthesis. (A)** Schematic of the *E. coli* replisome during the elongation step of an Okazaki fragment. **(B)** Lagging strand polymerase meets the RNA primer of the previous Okazaki fragment and stops synthesis. **(C)** Current model of events following completion of an Okazaki fragment. DNA polymerase is released from the β clamp (step 1) and the same molecule rebinds to a new β clamp to start the next Okazaki fragment (step 2). **(D)** An alternative model based on evidence from T4 and T7 replisomes. After completing the Okazaki fragment, the DNA polymerase detaches from the rest of the replisome (step 1). A new molecule of DNA polymerase is recruited to the replisome (step 2) and engages in the synthesis of a new Okazaki fragment. In this tentative model, a local pool of “spare” polymerases may facilitate their exchange and additional components may exchange along with the polymerase (not depicted).

Stability may be the result of the extended lifetime of some of the subassemblies of the active replisome. The idea of tight binding between its components is intimately linked to the trombone model of DNA replication. At its conception, this model arose to explain synchronous action of polymerases on both strands, and postulated a physical coupling between polymerases at the leading and lagging strands during synthesis. Importantly, it also suggested that the same copy of Pol III is recycled for multiple rounds of synthesis at the lagging strand (Sinha et al., 1980). Validation of this model came from experimental evidence showing that the replisome in *E. coli* can contain two polymerases (Maki et al., 1988; Onrust et al., 1995), and that they synthesize the two strands simultaneously (Yuzhakov et al., 1996). Long residence times of core replisome components have been demonstrated by ensemble and single-molecule *in vitro* studies of the *E. coli* replisome that show processive DNA synthesis in the absence of any free helicase, clamp loader or Pol III in the buffer (Li and Marians, 2000; Yao et al., 2009). From this data it is inferred that helicase, clamp loader and polymerases form a tight structure in the replisome, so that the same molecules of these proteins may be used over long periods, potentially even the whole replication event (Figures 3A–C).

However, this model of the replisome contrasts with evidence from T4 and T7 phages, which shows that the replicative polymerase is frequently replaced during fork progression (Yang et al., 2004; Johnson et al., 2007). Although the number and nature of their components differ, both of these systems have a similar architecture to the replisome of *E. coli*, indeed the trombone model was originally proposed for T4. Similarly to *E. coli*, coordination between leading and lagging strand synthesis has been extensively validated in these systems, and their replisomes can also carry out DNA synthesis in the absence of excess polymerase in the buffer (Debyser et al., 1994; Yang et al., 2004). Nevertheless, polymerase exchange can be detected shortly after addition of mutant polymerase to a reaction where the replisome is engaged in DNA synthesis (Yang et al., 2004; Johnson et al., 2007). How can we reconcile frequent polymerase turnover with efficient recycling on the lagging strand? At least in the case of the T7 replisome, which has been studied using elegant single-molecule studies, this seems to be achieved by creating a local pool of “spare” polymerases available for fast switching. The replisome in this phage consists of only four proteins: gp5, the DNA polymerase; gp4, which acts as helicase and primase; gp2.5, a ssDNA binding protein; and *E. coli*’s thioredoxin which serves as processivity factor for the polymerase (serving the same function as the β clamp in the bacterial replisome). The gp5 polymerase interacts directly with gp4, but importantly, these proteins have two modes of interaction resulting in tight or loose association, respectively (Hamdan et al., 2005; Kulczyk et al., 2012). The tight interaction has a greater contribution to the processivity of the polymerase, but the weak interaction additionally permits the presence of up to six polymerases at the replication fork (Loparo et al., 2011; Geertsema et al., 2014).

The dynamics of phage replisomes are relevant to *E. coli* since they both show different behaviors depending on the concentration of their components in solution. Studies performed *in vitro* in the absence of excess components show the *E. coli* replisome can function without exchange of its subunits, but it is currently unclear if a different behavior will be observed inside the cell (Figure 3D), where the diffusing pool of replisome components is one-to-two orders of magnitude higher than the number of active molecules (Leu et al., 2000; Reyes-Lamothe et al., 2010). It is easy to imagine how the capacity to detach polymerases from the replisome would be advantageous in order to rapidly respond to lesions and roadblocks on DNA. Multiple studies have shown that after encountering an obstacle, polymerase can unbind from DNA and subsequently re-engage via alternative mechanisms, leaving a gap in the double strand (Pomerantz and O’Donnell, 2008, 2010; Yeeles and Marians, 2011). The high processivity of polymerase bound to the β clamp conflicts with the observed “hopping” over obstacles on DNA, but complete detachment from the replisome would reconcile the long residence of polymerase on DNA with progression of the replication fork, potentially providing greater flexibility to the replisome.

In this scenario, replisome components otherwise thought to be stable are actually moving parts that actively exchange; one wonders what would then help maintain the stability of the replisome as an assembly. Reiterating its central role in the control of DNA replication, the most likely candidate would be DnaB helicase. Indeed, measurements on the processivity of the replisome show that helicase is its most stable component, although multiple contacts to DNA from engaged polymerases seem to also help increase the processivity of the replisome (Yao et al., 2009). Whatever strategy the replisome may use, work still needs to be done to shed light on the interplay between its dynamics and stability.

## Disassembly of the Replisome

A key mechanistic challenge faces the bacterial replisome during termination of DNA replication. The circular nature of the bacterial chromosome dictates that a pair of replisomes that initiate from a single origin of replication will eventually converge on each other in a head-to-head orientation. Positive supercoiling accumulates between the two replisomes as they converge, but the activity of DNA gyrase, which normally removes positive supercoils, becomes limited by the decreasing amount of template DNA available. Instead, supercoils may diffuse behind the replisomes, forming precatenanes between newly replicated DNA; in *E. coli* these must be resolved by Topoisomerase IV for chromosome segregation to occur (Wang et al., 2008). Alternatively, it has been shown *in vitro* that a combination of the 3′-5′ helicase RecQ and Topoisomerase III is sufficient to directly resolve topological stress ahead of converging replication forks, in the presence of SSB (Suski and Marians, 2008).

**FIGURE 4:**
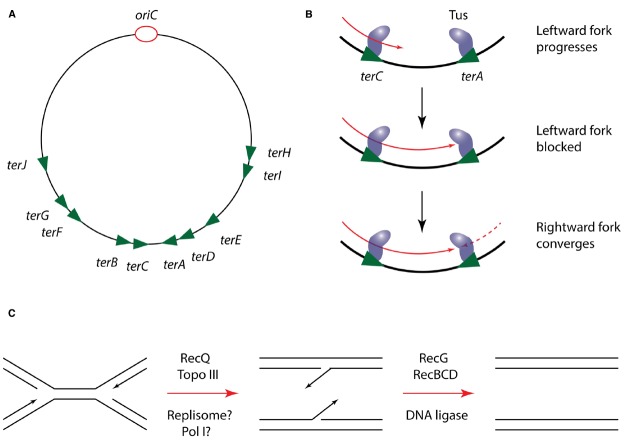
**Mechanism of replication termination in *E. coli*. (A)** Schematic of the *E. coli* chromosome showing the approximate location of *ter* sites relative to *oriC*. Arrowheads indicate the permitted direction of replisome progression. Adapted from Duggin et al., 2008. **(B)** Mechanism of replisome trapping in the terminus region by Tus-*ter*. Just two *ter* sites are shown for clarity but the same mechanism can operate at any *ter* site. **(C)** Resolution of DNA structures following replisome convergence during termination.

In at least some bacteria, the progression of replisomes is modulated to ensure that their convergence is restricted to a specific terminus region of the chromosome. This is best characterized in *E. coli*, where the Tus protein binds tightly to specific DNA sequences in the terminus, designated *ter* sites, and halts progression of the replisome. Crucially this effect is dependent on the orientation of *ter* sites, such that replisomes can enter but not exit the terminus region (Figure 4). The exact mechanism by which Tus-*ter* is able to block the replisome is still a matter of debate. Biochemical and structural studies have demonstrated that DnaB-catalyzed unwinding of *ter* DNA in the blocking orientation results in flipping of a specific cytosine into a binding pocket in Tus, strengthening its interaction with DNA and blocking further helicase progression. In contrast, unwinding from the opposing direction does not trigger this base flipping and results in displacement of the Tus protein from DNA (Neylon et al., 2000; Mulcair et al., 2006). However, an alternative model proposes that Tus mediates its effect on the replisome through direct protein-protein interactions with DnaB (Mulugu et al., 2001). This argument is supported by the fact that Tus can arrest DnaB translocation on dsDNA *in vitro* independently of *ter* unwinding (Bastia et al., 2008). However, given that DnaB within the replisome translocates on ssDNA, the relevance of this observation is unclear.

Regardless of the precise mechanism by which Tus operates, its regulation of the replisome does not appear to play a direct role in the process of termination, since it can be deleted from *E. coli* without producing a detectable phenotype (Roecklein et al., 1991). Furthermore, the Tus-*ter* system is poorly conserved among bacteria; while *B. subtilis* possesses a functional homolog of Tus (RTP) the two proteins lack any significant sequence or structural homology. The role of these replisome “traps” is thus more likely to be in coordinating termination with other processes localized to the terminus region of the chromosome, such as chromosome dimer resolution by XerCD (Duggin et al., 2008).

Genetic analysis suggests that the actual site of replication termination contains regions of overlapping DNA sequence that must be resolved into single daughter strands through the action of the RecG DNA translocase and the helicase-nuclease RecBCD (Rudolph et al., 2013; Wendel et al., 2014). Crucially however, it is unclear exactly how these termination structures are generated. Some analysis has suggested that DNA Pol I may play a role in replicating DNA at the terminus, but it is unclear if this occurs in coordination with the Pol III machinery or following its disassembly (Markovitz, 2005). Thus in particular, the role of the replisome in the final stages of chromosomal replication, and the fate of the replisome during termination are poorly understood.

Failure to disassemble replisomes in a timely manner is likely to result in genome instability; *in vitro* studies have demonstrated that *E. coli* replisomes can switch strands after converging upon each other, resulting in chromosome over-replication (Hiasa and Marians, 1994). However, it has not been addressed whether replisomes simply dissociate upon converging, or whether their disassembly is an active process. It has recently been shown that disassembly of the replisome in eukaryotic organisms is an active process, triggered by post-translational modification of the replicative helicase, Mcm2-7 (Maric et al., 2014; Moreno et al., 2014). Given the extensive regulation of bacterial helicase loading during initiation and replisome re-loading, it may be speculated that the bacterial replisome is subject to similar mechanisms of regulated disassembly.

## Future Perspectives

The information summarized in this work suggests that replisome organization and function follow a common theme with minor variations across bacteria, underlying the importance of DNA replication as a basic metabolic function of the cell. However, more work is needed in order to assess if these conclusions can be extended to all bacteria. Novel technologies in imaging, sequencing and genome editing will likely help us in the study of the replisome at a single-molecule level and *in vivo*, and to extend this analysis to non-classical model organisms. Future research promises a greater understanding of the composition of the replisome; how it is assembled; how it remains assembled; and how it comes apart. Furthermore, work in other bacteria will be especially revealing in understanding how specialized additions to the replisome help adapt organisms to their particular physiological needs. In turn, this information can catalyze the discovery of a set of basic considerations that any replisome must meet in order to achieve fast and efficient genome duplication.

### Conflict of Interest Statement

The authors declare that the research was conducted in the absence of any commercial or financial relationships that could be construed as a potential conflict of interest.
